# Insights into the utility of small form air quality monitoring in health care environments: lessons learned from the University Hospitals Birmingham NHS Foundation Trust

**DOI:** 10.3389/fpubh.2025.1634075

**Published:** 2025-08-26

**Authors:** Nicole Cowell, Clarissa Baldo, Kawun Williams, Catherine Muller, Siqi Hou, Daniel Rooney, Jian Zhong, Scarlett Healey, William James Bloss, Suzanne E. Bartington

**Affiliations:** ^1^Centre for Environmental Policy, Faculty of Natural Sciences, Imperial College London, London, United Kingdom; ^2^School of Geography, Environmental Sciences, University of Birmingham, Birmingham, United Kingdom; ^3^Univ Paris Est Creteil and Université Paris Cité, Paris, France; ^4^University Hospitals Birmingham NHS Foundation Trust, Birmingham, United Kingdom; ^5^Computational Science and Engineering Group, Centre for Advanced Simulation and Modelling, University of Greenwich, London, United Kingdom; ^6^Birmingham Women’s and Children’s NHS Foundation Trust, Birmingham, United Kingdom; ^7^Department of Applied Health Sciences, School of Health Sciences, College of Medicine and Health, University of Birmingham, Birmingham, United Kingdom

**Keywords:** air quality, sensors, diffusion tube, healthcare settings, NHS

## Abstract

Air pollution is a major environmental and public health challenge, with exposure to air pollution linked to 29,000–43,000 premature deaths annually in the UK. The National Health Service (NHS) experiences increased burden on its services due to air pollution related disease. Acute NHS Trusts and other healthcare settings are often locations with high inpatient and outpatient populations at enhanced vulnerability to air pollution related disease, including the young and older adults, and those with chronic health conditions. Many UK healthcare facilities are located in areas of poor air quality. Non-communicable disease from air pollution (PM_2.5_ and NO_2_) could cost health and social care providers estimated to >£18billion in the UK (between 2018 and 2035) if pollutant concentrations are not reduced. The NHS Long Term Plan recognised the need for NHS services to take action to mitigate air pollutant emissions, including those arising from site activities and patient, visitor and staff travel. However undertaking air quality monitoring and implementing targeted air-pollution interventions can present organisational, financial, and logistical challenges. Furthermore, evidence of the effectiveness of highly localised interventions is limited in the healthcare context. The recent expansion in utility of small form air quality sensors offers major potential to overcome some of the challenges in monitoring and understanding efficacy of targeted interventions at healthcare settings. Here, we present a case study from Queen Elizabeth Hospital Birmingham, a tertiary site managed by University Hospitals Birmingham NHS Foundation Trust. This study assesses the feasibility of small form monitoring (diffusion tubes and sensors) for evaluating local air quality interventions in healthcare settings, via an assessment of a localised traffic management scheme aiming to reduce local air pollutant concentrations.

## 1 Background

### 1.1 Air pollution in health care settings

There is a clear need to address air pollution in the UK. Deaths attributed to anthropogenic and natural sources of PM_2.5_ exceeded 8 million worldwide in 2021 (1) and exposure to air pollution is linked to 29,000–43,000 premature deaths annually in the UK (65). Air pollution related disease increases burden on healthcare services (5). Around 30% of preventable deaths in England are linked to non-communicable disease attributed to air pollution (2). The healthcare costs of poor air quality for NHS and social care providers in the UK could reach in excess of £18 billion (between 2018–2035) if concentrations of key pollutants are not decreased (3). In 2019 the NHS recognised that over 200 hospitals and 2000 GP practises nationwide were located in areas of air pollution concern for health (2), a figure which will likely increase with the (updated) World Health Organisation (WHO) Global Air Quality Guidelines in 2021. Tertiary hospitals which provide specialist and advanced treatments as major hubs for the treatment of patients with complex conditions including those particularly vulnerable to poor air quality, they often are located within densely populated urban areas that exceed legal air quality guidelines and WHO health-based recommendations.

Whilst the sources of air pollution at a tertiary hospital site are typically not constrained to service related NHS emissions (many will come from regional and other wider sources, such as traffic, industry and residential fuel combustion), NHS England has recognised its own emissions of air pollutants need addressing. The NHS Long Term Plan outlines key targets to cut vehicle fleet emissions by 2023/24 and includes an ambition for ≥90% of the NHS fleet to use low emission engines by 2028. Primary heating from coal and oil fuels is being phased out at NHS sites and there are ongoing advances in the adoption of virtual consultation and ward technology to further reduce staff and visitor mileage (2). Whilst NHS-wide actions like these are practical steps towards lowering pollutant emissions, additional actions can be taken by NHS trusts and healthcare workers themselves to support change (4).

Recent research has started to outline and evaluate effectiveness of local actions that can support air quality change for healthcare settings and their patients. Howard (4) suggested five ways GPs as individuals can take action to improve air quality, including educating themselves and patients about air pollution, encouraging active and public transport and joining campaign and awareness groups. Simpson et al. (5) evaluated the actions trusts can take to improve air quality and found that the most impactful short-term actions were shifts in energy production and transport strategy, whilst the most impactful long-term actions were communication and training, and effective leadership in sustainability practises. However, the most impactful actions were not always the most achievable, limiting their practise in already fiscally and operationally pressured healthcare settings. The air quality levels at healthcare sites are also likely to be impacted by sustainability actions and there is a need to ensure that any carbon mitigation actions to achieve mandated NHS England carbon emissions targets also deliver local air quality improvements (co-benefits) wherever possible. The potential of access to hyperlocal air quality data to support decision making may help in supporting uptake of actions that improve air quality, by providing evidence of air quality exceedance and improvement, and so providing a mechanism to incentivise behaviour and policy change. Further, pre-and post – intervention air quality data may be used to evaluate the effectiveness of localised actions and therefore strengthen the scientific evidence base.

### 1.2 Setting: queen Elizabeth hospital, Birmingham

University Hospitals Birmingham NHS Foundation Trust (UHB) is a large NHS trust comprising four acute hospital sites in the West Midlands, with over 20,000 staff, 2 million outpatient attendances and 370,000 admissions annually (6). The trust has an organisational commitment to improving its sustainability performance, with air quality and carbon emissions being a branch of this including the need to action the NHS Long Term plan. The UHB (7) Sustainability strategy recognised that staff commutes by car totalled over 85 million km a year, and whilst there has been a reduction in single occupancy car usage and an increase in active travel, there was still a need for further action (6). The trust plans to use a multi-faceted approach to improving air quality with a main focus on transport emissions as outlined in Table 1.

**Table 1:** The goals, actions and performance monitoring indicators for transport related air pollution and carbon mitigation at UHB sites (6, 64).

Goals	Actions	Monitoring of progress
Ensure significant proportion of outpatient appointments are virtual (online or phone) Reduce business travel across and between sites Reduce demand for car parking Reduce single occupancy car usage Increase the efficiency of deliveries to reduce carbon and air pollution emissions Increase the use of active travel by staff and patients Cut business mileage and vehicle fleet emissions by 20% by 2023/24 Reduce NO_2_ and PM concentrations at trust sites to fall inline with the national legal limit for NO_2_ and progress towards WHO guidelines for PM.	Continue to digitise the outpatients programme, shifting to virtual appointments when possible to reduce journeys Invest in teleconferencing, video and homeworking infrastructure Support the redevelopment of University train station (a station located at the edge of the QE hospital campus) Promote the use of alternate modes of transport to single occupancy car journeys to staff, including car sharing applications Continue to provide a staff shuttle service between sites Promote active travel by providing facilities such as lockers, changing and showers at sites Ensure any new fleet vehicles are electric or hybrid where possible Set lower emissions standards for external suppliers Roll out electric charging points for vehicles Improve sharing of public transport and air pollution information to staff and patients (for example, notes in appointment letters) Extend the travel survey to all sites to better understand staff and patient travel choices Evaluate and monitor against the Clean Air Hospital Framework Work with University of Birmingham (WM-Air project) to monitor air quality and identify areas suitable for intervention measures Raise awareness of air pollution by engaging in initiatives such as Clean Air Day	Regular travel survey across all sites to assess staff and patient travel choices Health outcomes travel tool Real-time air quality measurements on site Recording proportion of fleet that is electric or hybrid Recording the provision of electric vehicle infrastructure on sites Assess against Clean Air Hospital Framework Record proportion of video and telephone outpatient appointments

In this paper, we assess the feasibility of small-form air quality monitoring as a method for informing and assessing the effectiveness of targeted air quality interventions, using the UHB NHS foundation trust as a case study healthcare setting. This aligns with the monitoring progress action in Table 1“Real-time air quality measurements on site.”

### 1.3 Air quality monitoring in acute health care settings

The Clean Air Hospital Framework outlines air quality monitoring on hospital campuses as a recommended action for hospitals to both increase awareness and to feedback into decision making and target setting (8). Traditional methods of monitoring air quality are expensive (scale of £10,000’s–100,000’s per instrument, not including staffing), require specialist staff and can be time, energy and labour intensive. Therefore, it has been impractical to have long term, or continuous sampling of air quality at hospital campuses. In the UK, the Automatic Urban and Rural Network (AURN) is used for compliance checks against national air quality standards (9). Whilst there are sites in many major towns and cities, there are only 171 current sites across the UK, and they can often be located some distance from hospital settings. This means that they are not able to provide insight or evidence into the effectiveness of actions on reducing concentrations on the hospital campuses’ themselves but are rather reflective of regional concentrations. The development of ‘low-cost’ or small-form (£100–£5,000) sensors creates a novel opportunity for monitoring air pollution at higher spatial resolutions, including monitoring in hospital settings (10, 11). This current article reports detailed findings arising from fieldwork campaigns upon which the published guidance is based, considering the feasibility and scalability of hyperlocal air quality monitoring campaigns in acute trust settings. This paper presents a collaboration between an NHS Trust and academia, exploring the feasibility, validity and utility of air quality monitoring using small form sensors and diffusion tubes in healthcare settings.

#### 1.3.1 Diffusion tubes for NO_2_ assessment

Diffusion tubes offer an affordable (although less timely, and arguably less accurate or precise, than traditional monitors) insight into NO_2_ concentrations at a given location. Diffusion tubes are readily deployable by non-specialist staff, using passive sampling and utilise the principle of molecular diffusion. An absorbent substance located within the closed end of the tube absorbs ambient NO_2_ and this is used to calculate the average concentration of NO_2_ during tube exposure, via subsequent offline chemical analysis (12, 13).

Accredited laboratories offer a tube and analysis service, in which users simply deploy the tubes for a period at a location of interest and return via to the laboratory for analysis and results (14). As such, very little specialist air quality or related technical knowledge is required to carry out diffusion tube sampling. The tubes are unintrusive, require no power for sampling and are well suited to longer term monitoring, which is ideal when testing the effectiveness of an intervention compared to a baseline (15). It is recommended that each diffusion tube is deployed for a period of 2–4 weeks, making them suitable for insight into monthly concentrations [Gradko Environmental (7)]. Studies have shown that NO_2_ diffusion tubes report average concentrations within ±10–20% of reference air quality instruments, although this uncertainty increases when looking at individual tube measurements rather than averages (15, 16).

#### 1.3.2 Small-form sensors for particulate matter assessment

Small-form and ‘Internet-of-Things’ (IoT) enabled technology has created a paradigm shift in environmental monitoring in recent years. The recent growth of the small-form sensors for assessing environmental parameters and the rapidly evolving technology of wireless sensor networks means there is a growing market of affordable and “wireless” air quality monitoring technology (17, 18). Sensor networks are growing in popularity due to their relatively low equipment costs compared to regulatory sensing (£10’s–1000’s per unit rather than £10,000 + per unit) and their reduced need for specialised staff to manage them (19). These units can be self-contained, using battery or solar power, and are often designed to be plug and play for ease of consumer use. As such, they present a great opportunity for providing affordable and flexible continuous real-time air quality monitoring at healthcare sites.

Whilst small-form sensors for gaseous pollutants exist, the most promising performance comes from low-cost particulate matter (PM) samplers which have been shown in many studies to be successful in recording indicative PM concentrations in various settings and in some cases, have been granted MCERTS accreditation (17, 20–26). Low-cost PM samplers tend to refer to an Optical Particle Counter (OPC) approach, which uses light scattering technology to detect particles pulled through a sampling chamber and convert particle numbers into concentration. Whilst OPCs have been successfully used in indicative sampling, there is some uncertainty associated in the concentrations detected compared to reference grade instruments (such as gravimetric samplers). Humidity, the composition (hydroscopicity) of particulates and the environment sampling in can all affect the performance of a sensor and it is important that low-cost sensors are calibrated in environments with similar particle compositions and meteorology to which they will be sampling in to capture the effect of such variables on sensor performance (23, 27–29).

Some plug and play sensors designed for use in non-research settings are calibrated by the manufacturer. Whilst this makes the units easy to use and deploy without the specialist scientific skills required for calibration, it also presents a methodological challenge as often these calibration methods are not disclosed and are typically retained as intellectual property of the manufacturer. This means it is not always clear how the raw data has been manipulated ahead of being received by the user (11). Therefore, it is important to also consider a validation process for low-cost sensor data to ensure data quality (30). There are examples of data validation methods from the literature which could easily be applied to a dataset ahead of any analysis, generally they factor in manufacturer stated limitations, periods of static data and completeness criteria (31–33).

There is a clear need for further understanding of air pollution in healthcare environments. Hospitals and other healthcare services are frequented by some of the most vulnerable populations to the impacts of air quality and patients and staff are frequently exposed to concentrations which are recognised as hazardous to health. Small-form sensors and diffusion tubes provide a potential practical solution to undertaking air quality monitoring in healthcare environments, which could in turn provide a mechanism for planning and assessing targeted local interventions. However, the feasibility of adoption of this approach to local data capture and use to support decision-making remains uncertain. This case study at a major acute NHS trust therefore assessed the real-world application of such devices, assessing potential to inform and support decision making and assess changes related to local interventions.

## 2 Methods

### 2.1 Sampling context

The largest acute hospital site operated by the trust is the 1,215 bed Queen Elizabeth Hospital Birmingham (QEHB) (34). The hospital is located in the Edgbaston suburb of Birmingham about 4.5 km southwest of the city centre. The hospital campus is large, compromised of multiple patient care, research and teaching buildings across an area of approximately 0.3 km^2^ with multiple roads and carparks within the campus bounds. This hospital is well placed for encouraging transport modal shift, as it has direct access to the adjacent University train station, canal cycleways and bus routes serving the hospital. Between the 2021 and 2023 sampling campaigns, QEHB implemented traffic management changes to the Main Entrance loop by introducing a bus lane with the aim of reducing congestion. From personal correspondence, the bus lane removed 5 parking spaces to allow room. This allows buses to not be held in congestion and reduces the area available for idling parked cars, however, does not affect the congestion of traffic other than buses on the rest of the loop.

UHB works closely alongside a second NHS trust in the Birmingham area, Birmingham Women’s and Children’s NHS Foundation Trust (BWCNHSFT). BWCNHSFT was the first hospital trust in the UK to focus on providing healthcare solely to families, women and children providing specialist treatment in paediatric, neo-natal and foetal medicine. The trust has 2 main hospital sites. Birmingham Women’s Hospital is located in its own building within the QEHB campus and provides services to over 50,000 women and delivers >8,000 babies annually (35). The QEHB campus was undergoing construction at the time of this project, with construction in various sites across the campus. Birmingham Children’s Hospital is located within the city centre and within the Birmingham Clean Air Zone, adjacent to the A38, a major traffic route passing through the city (36). BWCNHSFT also has a Sustainability strategy, or “Green Plan” focusing on net zero actions. There are often positive relationships between actions for net zero and improvements for air quality and this plan does recognise some of the co-benefits of actions such as fleet changes and transport modal shift for air pollution (37).

### 2.2 Data collection: sampling locations

NO_2_ and PM were chosen for sampling as these are the pollutants associated with significant health risks and are the current focus of local air quality interventions (such as Birmingham’s Clean Air Zone for NO_2_). Prior research has shown that concentrations for both pollutants exceed the WHO annual guidelines in the Birmingham area (10 μgm^−3^ for NO_2_, 5 μgm^−3^ for PM_2.5_) (38, 39).

**Figure 1:**
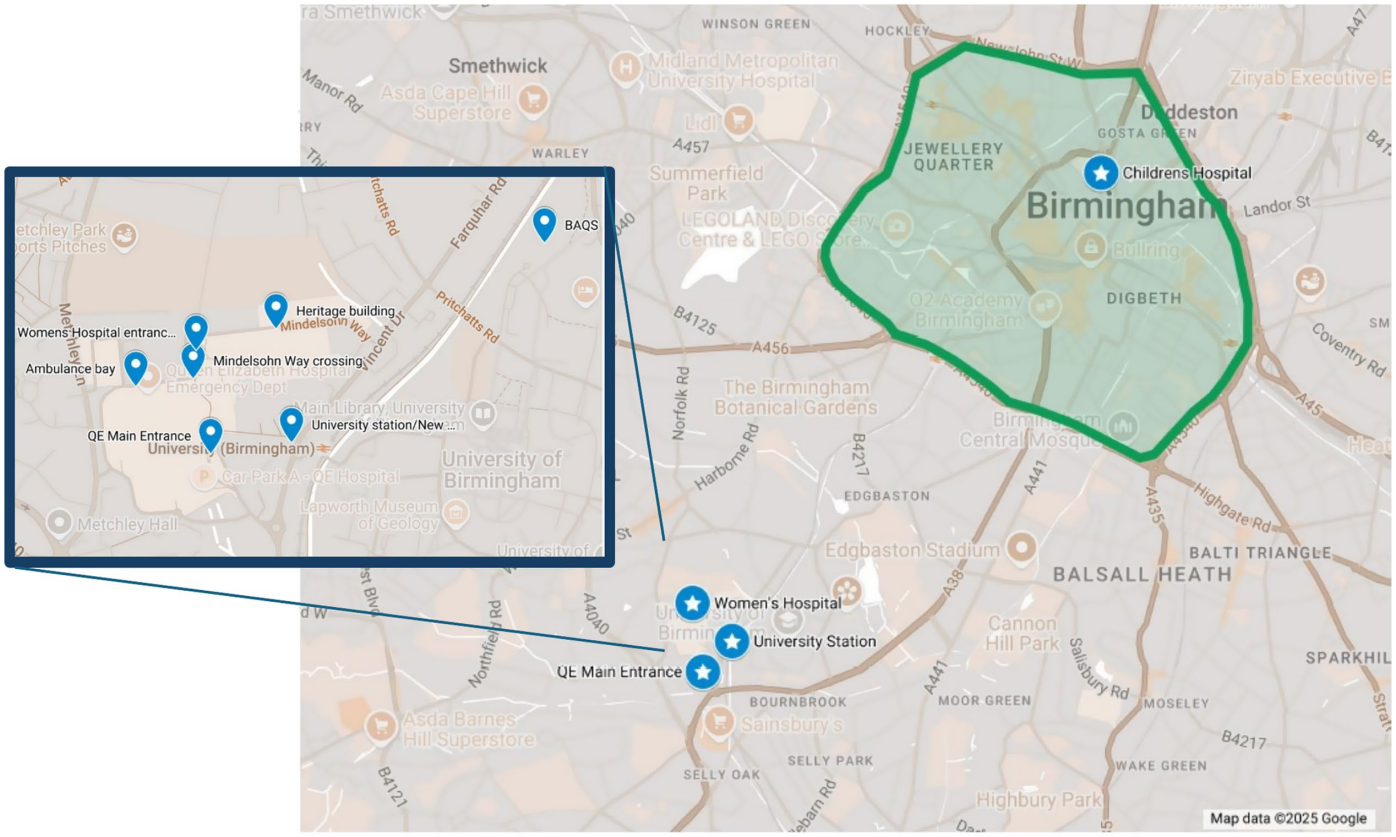
Map showing the sampling locations for diffusion tubes and low-cost PM samplers across the hospital sites in Edgbaston, Birmingham (UK). Star points show the low-cost PM samplers and dot points in the highlighted inset show the zoomed in view of the diffusion tube sampling locations at QEHB hospital campus. Green zone shows the Birmingham Clean Air Zone. Map created using Google Maps (Map data ©2025 Google).

The sampling locations and rationale for selection are shown in Figure 1 and Table 2. Locations were identified in collaboration with the sustainability teams and public health consultants at UHB and BWCNHSFT and were chosen to target areas of pollution concern (due to construction, vehicles idling etc); areas of high patient exposure (entrances, pedestrian areas); and areas of planned air quality interventions (such as the bus lane intervention above). We were unable to mount sensors or diffusion tubes on buildings due to legal constraints for building maintenance under public finance initiative (PFI) contractual obligations. Instead existing street furniture (lamp posts/signposts) were used. Diffusion tube (NO_2_) sampling was carried out at the QEHB campus only, whereas PM sampling occurred across both UHB and BWCNHSFT sites.

**Table 2:** Location description and rationale for diffusion tube (NO_2_) and PM sampling.

Sampling location name	Location description	Which trust is the location situated at?	Sampling type	Rationale
QE Main Entrance	On a lamppost within the drop off loop road for vehicles	QEHB	Diffusion tube and PM sampling	This is the drop off area for taxis, private vehicles and some public transport which is prone to idling vehicles and congestion. There is also a lot of pedestrian activity here as there are benches, smoking areas, fruit market and the main entrance to the hospital.
University Station	Directly opposite the entrance to the train station on the hospital campus	University of Birmingham campus	PM sampling	This location is directly outside of the train station which serves the University and QEHB. A small access road to the University and a pedestrian route are next to this sensor.
Women’s Hospital	On a lamppost at the entrance to the hospital building and carpark. Next to a pedestrian waiting area.	BWC	Diffusion tube and PM sampling	Entrance to car park and drop off area so concern around idling vehicles. Outside benches and patient waiting area nearby.
Children’s Hospital	On the front façade of the building, facing the road.	BWC	PM sampling	The Children’s hospital is located close to the A38, a major road which often faces congestion. The façade was one of the few suitable locations available for sampling at this site. It is above the entrance area to the hospital.
BAQS	Urban background sampling site on the University Campus	University of Birmingham Campus	Diffusion tubes	Birmingham Air Quality Supersite (BAQS) is a research site on the University of Birmingham campus monitoring multiple air pollutants using reference grade equipment. This allows for a colocation and testing of diffusion tubes.
University Station New Fosse Way	New Fosse Way opposite University Train Station	QEHB	Diffusion tubes	This is near to the bus stops and train station on a busy road, on the pedestrian route that follows towards the QEHB main entrance.
Mindelsohn Way Crossing		QEHB	Diffusion tubes	Zebra crossing across road on pedestrian through route between hospital buildings.
Ambulance Bay		QEHB	Diffusion tubes	Zebra crossing in front of the ambulance drop off and waiting bay. Concerns raised by UHB around ambulance idling.
Heritage building		QEHB	Diffusion tubes	Located on the traffic route through the hospital campus, near to the multi-storey car park for visitors.

### 2.3 NO_2_ assessment: diffusion tubes

Diffusion tubes were deployed across the locations for a one-year period of baseline data collection (August 2020–July 2021) and a subsequent 4 month period in 2023 (April–July). The 2023 sampling was implemented to generate insights into whether interventions and changes made across the hospital trust site led to changes from the baseline concentrations in 2020–2021. Tubes were deployed in triplicates, 3 at each sampling location were used to generate an average concentration. Each deployment lasted for 4 weeks, with efforts made to ensure the sampling period provided the best monthly coverage, with data assigned to the month it best represented by the respective 4-week duration. The absorbent in the diffusion tube was 20% triethanolamine/de-ionised water. The deployment and collection time and date were recorded and shared with the laboratory for generating average concentrations. Tubes (nitrogen dioxide diffusion tubes, DIF100-20) were purchased from Gradko and returned to them for assessment by their laboratory with concentrations returned in average μgm^−3^ for the deployment period. Concentrations are generated by their laboratory team using a UV/visible spectrophotometry method, referencing a calibration curve for nitrate solutions that is UKAS accredited (40). Any storage of tubes before and after sampling was kept minimal, and tubes were stored with their caps on in sealed storage bags in a fridge.

### 2.4 PM sampling: air quality sensors

As part of a wider PM sampling campaign using ‘low-cost’ sensing equipment administered by the Birmingham Urban Observatory, Earthsense Zephyr air quality sensors were installed at 4 sampling locations (Table 2 describes sampling locations). For PM sampling, the Earthsense Zephyr houses a Plantower PMS5003. The Plantower is a OPC commonly used with low-cost samplers and has been extensively documented in within recent literature (19, 22, 23, 41–47). Generally findings from tests of the Plantower find that they can perform well against reference instruments, if corrected for the impact of humidity and composition and that sensors are consistent between themselves (23). There have been reports of the Plantower being ineffective at measuring PM_10_ due to particle loss during sampling and laser geometry, however in February 2023 Earthsense received MCERTS status for indicative monitoring of PM_2.5_ and PM_10_ using the Plantower OPC (24, 42, 44, 45). Earthsense provide their own calibrations to sensor data, which are undisclosed. The method for the online calibrations was updated in line with their MCERTS certification, although data used in this study was downloaded prior to this update. Discussions with the manufacturer confirmed that all Earthsense units undergo a co-location with a reference instrument in the UK and that this helps inform the calibration process which also accounts for the impact of humidity, although we are uncertain whether this is a static calibration or agile. This could impact the reliability of results as the specifics of how the data has been processed by the manufacturer platform before downloading and how this may change with time are not disclosed. Calibration is carried out in a different location to where the sampling takes place, thus it is not specific to the particle composition encountered at the measurement location (which may differ in hygroscopicity). At the time of writing, Earthsense report accuracy of 5 μgm^−3^ for PM measurements and a limit of detection of 2 μgm^−3^ and 5 μgm^−3^ for PM_1_ and PM_2.5_, respectively, (48). Sensor data used in this study were collected from 1st September 2021-31st August 2022, downloaded as 15-min averages and converted into hourly averages during data processing.

Data validation is also an important step in ensuring data quality when working with small form sensors. As these sensors were deployed as part of a wider project, a sensor validation process was established for all data (19) with results given in (49). This method draws from Lu et al. (32), Mousavi and Wu (33) and Bush et al. (31) and addresses measurement timeseries gaps/completeness, periods of little or no change, extreme outliers and the impact of meteorology. We used meteorology data from the Birmingham Air Quality Supersite, which is located close to the UHB campus for this step, although recognise that not all hospitals have access to such sites for data. Alternatively, many commercial small form sensors have the option for meteorology data, there are also small form meteorology sensors readily available commercially and open access meteorology data sets online for many cities. It is important to note that whilst these steps aim to address common challenges of small form sensors, there is a risk that some non-erroneous data may be filtered by such steps (true extreme peaks or periods of little change may be detected as outliers and removed). It is important that any future users consider this before applying validation and think about their sampling aims and expectations before selecting validation criteria. The 4 validation steps are outlined below:

*Step 1–75% completeness criteria* 3 or more measurements an hour (sensors measure every 15 min), 18 h or more coverage a day, 23 days or more coverage a month cumulative. Hours/days/months that fell outside of these criteria were discarded.
*Step 2- Meteorology filter* Keep data that falls within the confines of manufacturer specifications −10°C < t < 35°C and RH>35% using temperature and humidity data from the Birmingham Air Quality Supersite (BAQS) located on the University of Birmingham campus.
*Step 3- Static data* Remove data with 5 h moving standard deviation = 0.
*Step 4-Exclude outliers* Exclude data based on a threshold defined from 3x the Median Absolute Deviation as shown below in equation 1 & 2 drawn from Lu et al. (7), where $X_{i}$ is the PM_2.5_ reading of the sensor, $\tilde{X}$ is the median of $X_{i}$ in a month and b =1.4826 (a set constant).

$$\mathrm{MAD}=b*\text{median}(X_{i}-\tilde{X})$$

$$X_{i}<\tilde{X}-3*\mathrm{MAD}\;\text{or}\;X_{i}<\tilde{X}+3*\mathrm{MAD}$$

The data capture of the year period after data validation is displayed in Table 3.

**Table 3:** % capture of potential readings of PM_2.5_ (8,760 potential 1 h average readings within 1 year period) by sensor after data validation process.

Sampling location	% data capture between 1st September 2021-1st August 2022
Children’s Hospital	92
Women’s Hospital	76
QE Main Entrance	76
University Station	69

Data analysis weas conducted using Microsoft Office and R studio. The OpenAir package supported data analysis and visualisations when using R (50, 51).

### 2.5 Context of the COVID-19 pandemic

Sampling took place in August 2020–July 2021 for NO_2_ (diffusion tubes) and September 2021–August 2022 for PM_2.5_ (Zephyrs). During 2020 and 2021, the COVID-19 pandemic and the associated public health restrictions caused major changes to public behaviour and healthcare provision, including restrictions on site activities. The impacts upon pollutant emissions and concentrations in urban areas have been extensively studied including the application of methodological techniques to adjust for meteorological influences and ongoing trends, with published studies suggesting abrupt reductions in NO_2_ concentrations with limited impact on PM_2.5_ in urban areas, including Birmingham (52). Data used in the following analysis therefore mostly focusses on the spring and summer months of 2021 and 2023, avoiding the periods of national lockdown restrictions in the UK which were accompanied by major traffic reductions. By April 2021, non-essential business was restarting and from May 2021 most restrictions on social mixing where relaxed. Whilst we cannot eradicate the impact of the pandemic, from personal correspondence with the trust, we can establish that from spring 2021 the hospital was open to the public for appointments and was past the most restrictive parts of the pandemic. We are also aware of the shift to some outpatient appointments being held virtually in 2021, compared to pre-pandemic. For PM data, we can also acknowledge that a study into the impact of the COVID-19 lockdowns on PM concentrations across Birmingham found that in March–May 2020 the reduction of PM_2.5_ was limited (52, 53).

## 3 Results

### 3.1 NO_2_ concentrations

Although 1 year of air pollution data was collected in 2020–21, only 4 months of data are available in 2023. Therefore, our NO_2_ analysis focuses on the months April, May and June as where data is available at baseline and follow-up, enabling comparisons of data obtained during similar meteorological seasons. Table 4 demonstrates average meteorological conditions at the nearby Elms Cottage Weather Station on the University of Birmingham Campus for the various months of the study. Humidity was similar across the 2 sampling years, although temperature in 2023 was generally warmer than in 2021. Whilst meteorology and other factors (such as a return to activity from the COVID-19 pandemic) will have differed between the 2 years, all tubes are located in close proximity and subject to the same changes, so relative differences between sampling locations can be assessed.

**Table 4:** Meteorology monthly averages from elms cottage weather station on the university campus, located ~130 m away from Birmingham air quality supersite (BAQS).

Month	Year	Temperature °C	Relative humidity %
April	2021	5.6	68.2
	2023	9.9	76.9
May	2021	10.5	79.0
	2023	15.1	74.1
June	2021	15.9	74.6
	2023	19.5	70.0
July	2021	18.0	77.2
	2023	17.4	

A colocation of diffusion tubes against a reference instrument was carried out to test the accuracy of the diffusion tubes (Table 5). Literature suggests diffusion tubes are generally expected to have an uncertainty of ±20% if used correctly (16). When comparing the data from our on campus diffusion tube sampling at BAQS against the nearest DEFRA AURN urban background monitor (Birmingham Ladywood, approximately 3 km away), we found % accuracy ranged from −10.23 – + 11.02%.

**Table 5:** Comparison of data from the triplicate tubes at the BAQS location (urban background) against the nearest DEFRA AURN urban background monitor at Ladywood.

Date period	Ladywood DEFRA AURN NO_2_ concentration (μgm^−3^)	Average NO_2_ concentration from triplicate diffusion tubes at BAQS urban background location (μgm^−3^)	% accuracy
Apr-21	20.4	18.3	−10.2
May-21	13.1	14.5	11.0
Jun-21	11.4	12.5	10.2
Jul-21	11.7		
Apr-23	16.3		
May-23	14.1	13.2	−6.7
Jun-23	11.2	11.6	3.3
Jul-23	7.6		

**Figure 2:**
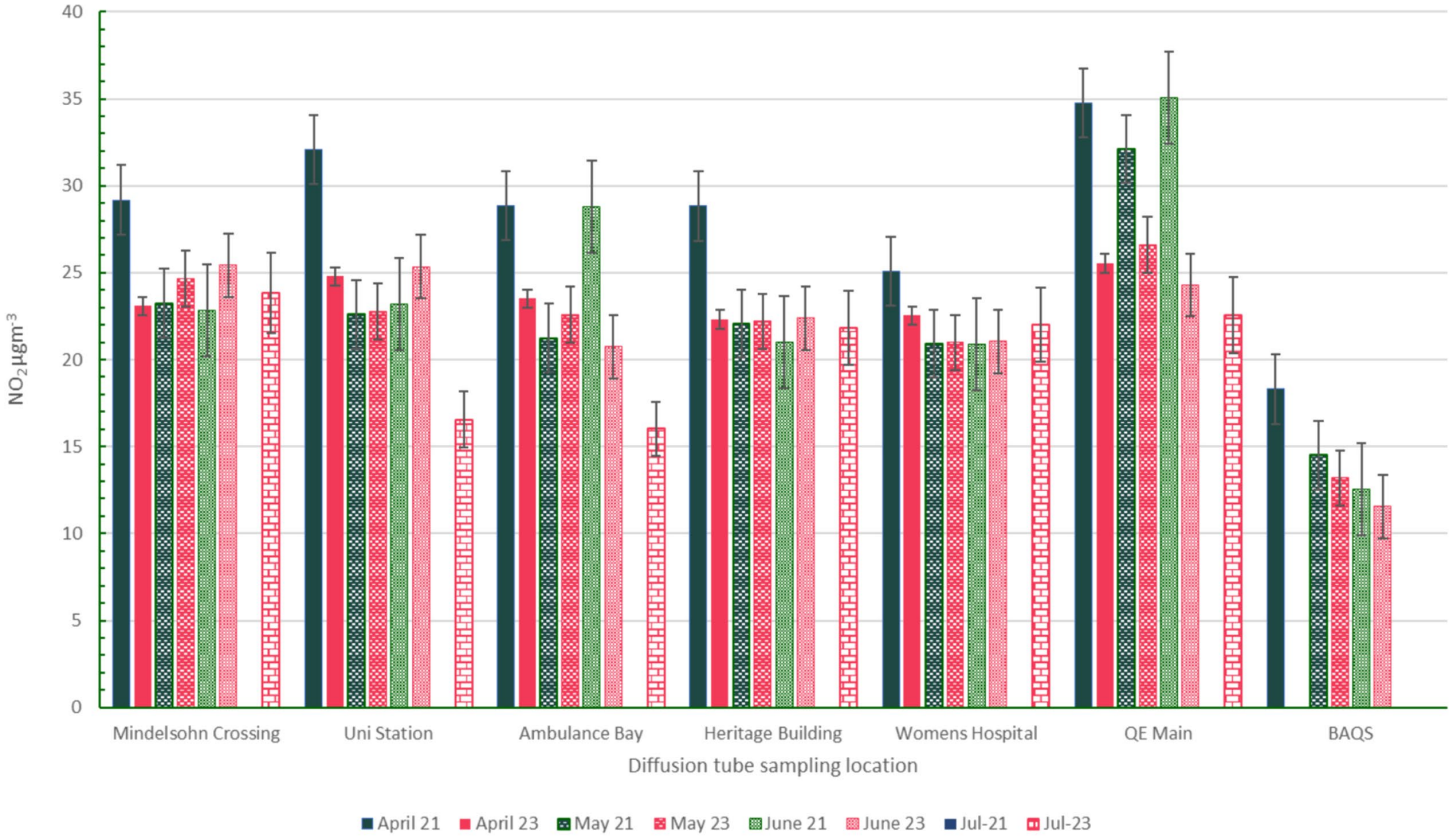
Bar chart of concentrations by month across all locations. Pink bars refer to 2023 whereas dark green bars refer to 2021.

All locations exceeded the WHO air quality guidelines for annual average NO_2_ concentrations (10 μgm^−3^) in both 2021 and 2023. Although monthly values for all sampling locations apart from Women’s Hospital and BAQS exceeded the daily average WHO NO_2_ guideline (25 μgm^−3^) in 2021, a limitation of the diffusion tube methodology is that we cannot infer daily averages. Table 6 suggests that QE Main Entrance experiences the highest concentrations of NO_2._ This is also reflected in monthly averages, with QE Main Entrance recording the highest concentrations across all but two of the months included in the analysis (see Figure 2 for months of data featured in this study).

**Table 6:** Average diffusion tube NO_2_ (μgm^−3^) concentrations of 4-month sampling periods in 2021 and 2023 by location.

Year	Mindelsohn crossing	Uni station	Ambulance bay	Heritage building	Women’s hospital	QE main	BAQS
2021 average	25.1	26.0	26.3	24.0	22.3	34.0	15.1
2023 average	24.4	24.3	22.3	22.3	21.5	25.5	12.4

**Figure 3:**
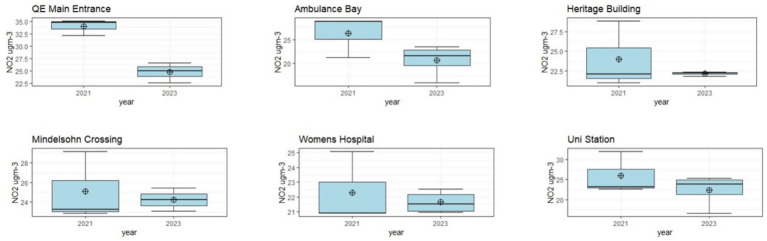
Boxplots comparing NO_2_ concentrations between the 2021 and 2023 datasets by location.

For all sampling locations, it appears that April 2021 was an episode of high concentrations at all sampling locations. Apart from this month, most locations (apart from QE Main Entrance) appear to have fluctuating concentration changes between 2021 and 2023 with no clear pattern of reduction. An exception to this is QE Main Entrance, which has statistically significant monthly NO_2_ concentration reductions between 2021 and 2023. A t-test shows that this is the only location with statistically significant difference between the baseline and follow-up datasets (*p* value <0.001, with a 95% ci that the difference between the 2021 and 2023 is between 5.9–12.6 μgm^−3^). As this is the only location where this pattern is observed we can infer this is unlikely to be attributable to influences of meteorology, and could be due to reduced congestion (either from a traffic intervention implemented here, or from the pandemic affecting traffic patterns). Further data could provide a more conclusive evaluation of the reduction in NO_2_ at this location (see section 4.3) Figure 3 demonstrates how clearly different the 2021 and 2023 concentrations at QE Main Entrance are compared to the other locations included in the campaign.

### 3.2 PM_2.5_ concentrations

Overall annual average PM_2.5_ concentrations were between 6.7 μgm^−3^ (BCH) and 7.8 μgm^−3^ (BWH) (Table 7). Annual average PM_2.5_ concentrations showed only 1.15 μgm^−3^ variation between locations, which is smaller than the suggested limit of detection indicated in past studies into the Plantower PMS5003 and therefore we cannot draw conclusions regarding differences in the annual averages between the sampling locations (23). All locations exceeded the WHO air quality guideline for annual average PM_2.5_ of 5 μgm^−3^, and the daily (24-h) average guideline of 15 μgm^−3^ was exceeded at times across all locations (although it is important to note that sensors may not be sensitive enough to reliably detect concentrations below the annual guideline concentrations) (38). The two locations on the QEHB campus (Women’s and QE Main Entrance) had the most exceedances of daily average guidelines (15 μgm^−3^), with 9.1 and 8.2% of measured days exceeding the daily guideline, respectively. QEHB campus has had periods of construction and regeneration of buildings during the sampling campaign, which may explain why there are more frequent exceedances at these sampling locations.

**Table 7:** Summary statistics and insight into WHO air quality guideline exceedances for PM_2.5_ from the Earthsense Zephyrs for the 1 year study period 1st September 2021–31st August 2022.

Location	Mean (μgm^−3^)	Max (μgm^−3^)	95th percentile (μgm^−3^)	25th percentile (μgm^−3^)	Median (μgm^−3^)	% of days recorded where daily (24-h) average exceeded WHO 24-h average guidelines (15μgm^−3^)
Children’s hospital	6.7	38.6	17.1	3.2	5.5	5.6
Women’s hospital	7.8	44.2	20.3	3.6	6.2	9.1
QE main entrance	7.6	37.1	20.3	3.3	6.3	8.3
University station	6.9	26.4	15.8	3.6	6	4.1

**Figure 4:**
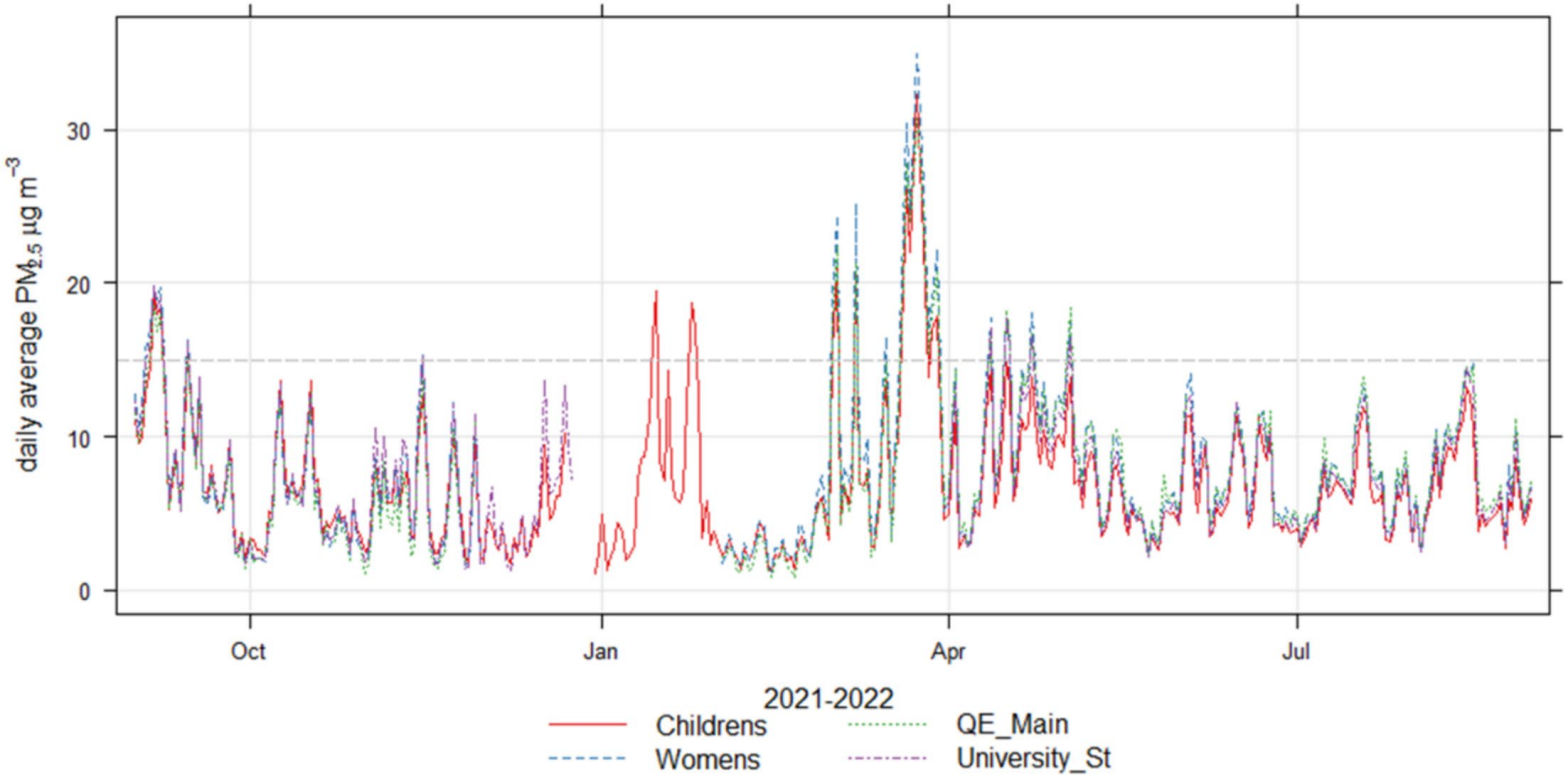
Time series plot of daily average PM_2.5_ concentrations across the locations from 1st September 2021 to 31st August 2022, with the grey dashed line demonstrating the WHO guidelines for daily average concentrations (15 μgm^−3^).

**Figure 5:**
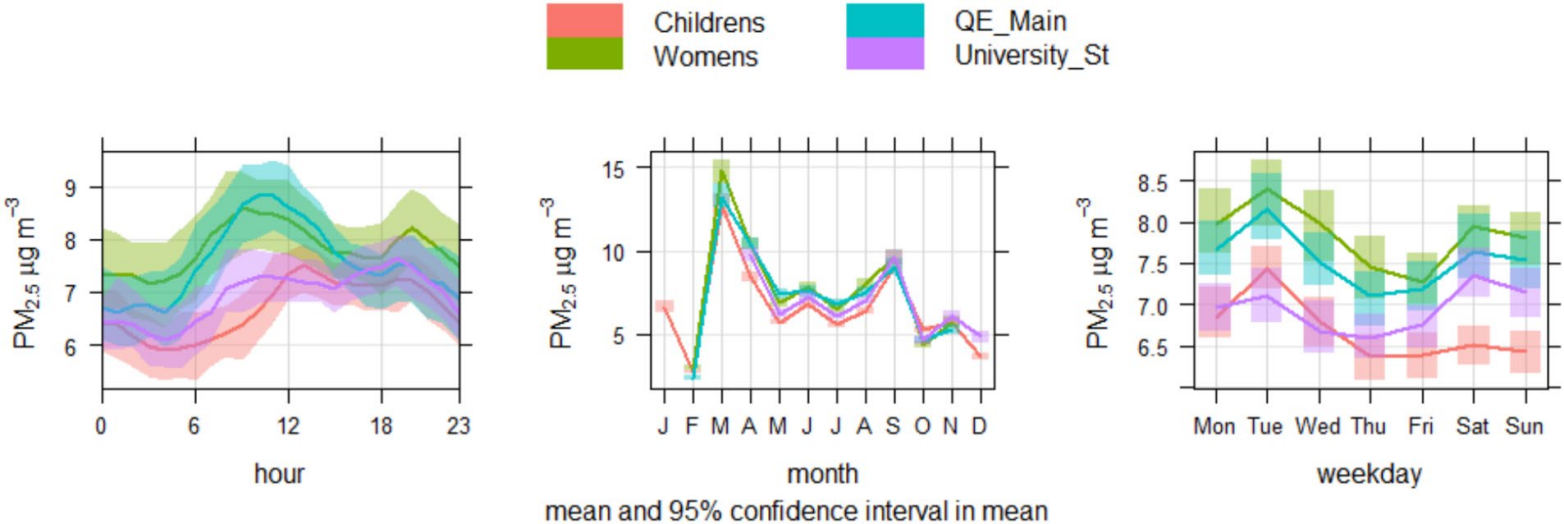
Time variation plots for PM_2.5_ concentrations, showing the average diurnal, monthly and weekly profiles across each location.

Figure 4 demonstrates the daily average concentrations across the year period and Figure 5 demonstrates the annual, diurnal and weekly patterns detected across the locations. The concentrations across all locations are well correlated: Pearson’s R ranges from 0.95–0.98 between locations. It is noted that all locations exhibited peaks in concentrations at similar times, suggesting regional PM events. Diurnal patterns appear to form 2 groups: QE Main Entrance and the Women’s hospital generally have elevated morning concentrations compared to University Station and the Children’s hospital. This could be associated with their location within close proximity of large carparks. Across all locations, there appears to be a weekly pattern of elevated concentrations on Tuesdays, a pattern which is also recognised in data available from the against the local DEFRA AURN roadside monitoring station (Birmingham A4540) show that this also reflects this pattern for PM_2.5_ concentrations during the same period, but not for NO_2_. This shows that this is a regional phenomenon, but that there is not a clear link with NO_2,_ which suggests that the PM peaks may not be related to traffic emissions.

## 4 Discussion

The concentrations of PM_2.5_ and NO_2_ at the hospital sites recorded by the indicative measurements suggest that there would be air pollution linked health risks to patients, staff and visitors. For both PM_2.5_ and NO_2_, measurements at all sampling locations exceeded the WHO annual average guidelines, which in the context of the COMEAP advice around long term exposure, suggests that those frequently in these areas are potentially going to experience elevated risk of air pollution related disease (7, 38). This demonstrates a need for air quality interventions which will support the healthcare settings in reducing exposure of vulnerable individuals to elevated concentrations. Whilst there are attempts to reduce NO_2_ concentrations elsewhere in the city in the form of the Birmingham Clean Air Zone (CAZ), the QEHB campus is not captured within the zone itself. Only sampling at the BCH was within the CAZ. It is also important to note that the CAZ focuses on the reduction of NO_2_ and evidence suggests that schemes such as CAZ and ULEZ have limited impact on PM concentrations with a recent study confirming no significant impacts of CAZ on PM_2.5_ in Birmingham (54–56). As PM concentrations contain significant background or regional components, to tackle PM concentrations at hospital sites both local and regional sources will need to be considered. Hospitals can work with their local governing bodies to encourage and advocate for air quality action to tackle PM across the region, which will not only reduce local exposure, but also decrease the strain on hospitals from PM related admissions of non-communicable disease.

### 4.1 The feasibility of air quality monitoring

We have demonstrated that passive air quality sampling via methods such as diffusion tubes should be practically feasible for long term monitoring at a healthcare setting. Within this study, both University and hospital staff were involved in the practical aspects involving changing of diffusion tubes as the methodology of simply switching tubes and posting to a lab for analysis was straightforward, rapid and did not require specialist skills. The work of changing diffusion tubes took ~1 h every 4 weeks and therefore should be considered feasible in terms of staffing time and resource allocation for healthcare settings to achieve. From our collaboration with the UHB sustainability team, we were also able to provide expert guidance on practise and sampling design, including triplicate tubes and sampling frequency, available at www.wm-airg.org.uk (10). Diffusion tubes have already been demonstrated successfully in community led monitoring at supplementing local air quality data (57), however it is important to ensure that users are aware of the caveats of passive sampling data. Whilst tubes may be able to highlight potential areas of concern or improvement, they are considered indicative measurements and thus have associated error (literature suggested ±10–20% compared to a reference instrument, which aligns with our error values of −10.23–11.02%) (12, 16). Therefore, assessments using diffusion tubes are most suitable to initial screening and assessing relative changes following local interventions (58). They are less well suited to providing absolute air pollutant concentration values or for regulatory equivalent monitoring. The overall expenditure on NO_2_ diffusion tube monitoring was approximately £7 per tube per month (for tube and analysis, not inclusive of shipping and clips to hold the tubes).

We identified that small-form sensors are slightly more challenging than diffusion tubes to introduce into healthcare environments in their current operational context. Whilst initial costs may be attractive to healthcare teams (and much lower than the regulatory instrument equivalent), the ongoing costs associated with maintaining a network and undertaking data processing can be harder to secure. With sensing-as-a-service units, funding is required both for capital purchase and also an annual data subscription fee to retain the unit on an online platform for data access. Costs therefore go beyond the initial set up, may be unforeseen at the campaign outset, and the lifetime or long-term maintenance requirements of many small-form sensors is still unknown (11). Beyond funding, in this study we had experienced researchers with expert knowledge of small-form sensors and the potential data challenges they pose (for example: bias, uncertainty, calibration) (59). Without this knowledge, and data processing skills there is a risk that data may be misinterpreted. Whilst sensing as a service offers the chance for non-specialists to outsource more technical skills such as calibration, this then can lead to challenges in data reliability if the data processing is not transparent.

### 4.2 Lessons learned for air quality evaluation of healthcare interventions

With so many bodies acting within healthcare settings, it is very easy for interventions that may impact air quality to occur in the absence of full documentation to support any sustainability-based decision making. The QE Main Entrance and bus loop is an example of this - with changing staff and many parties involved, it was challenging to gain full detail. To fully assess the impact of any interventions, it is important to also have access to supporting data beyond the air quality concentrations such as traffic counts, meteorology or other source/behaviour characteristics that may influence the outcomes. A full mixed- methods evaluation could also include qualitative data to better understand how people (including staff, patients and visitors in this context) respond to such changes.

However, combined with the demands on NHS sustainability teams to be meeting many goals at once (net zero, energy efficiency, waste management etc) and there are challenges to overcome in fully understanding air quality in such settings. There could be room to explore the role of co-benefits of air quality action to support the uptake of interventions and monitoring. Systems thinking and co-production with stakeholders has been recognised as an opportunity to overcome complexity of such challenges, to help integrate health into climate and sustainability decision making (60). Many air quality interventions align with reducing carbon emissions and therefore can support climate strategy simultaneously. By framing actions in this way, they may gain more traction and resource as different sustainability projects can pool resources for co-beneficial action.

Currently, for such monitoring work to be successful it is likely that collaboration with external partners will be key in supporting the most effective air quality monitoring and intervention development. Whilst consultancies are increasingly specialising in this area, academic partnerships offer potential here. Trust partnerships with Universities allow for transparency, open access data and the development of research and sustainability initiatives simultaneously. Lessons learnt from this project have highlighted the potential and challenges of research-healthcare initiatives. Dialogue with the trust allowed research to be tailored to support impact; identifying the agenda and concerns of sustainability and public health experts within the health care system. However, we also identified a disconnect between different departments and activities on the hospital campuses. For example, sustainability teams do not manage the transport data collected on site, despite transport pollution emissions being a concern of the sustainability team. This is not an uncommon problem; siloed thinking has been highlighted as a challenge in environmental decision making previously (61, 62).

### 4.3 Impact of interventions

**Figure 6:**
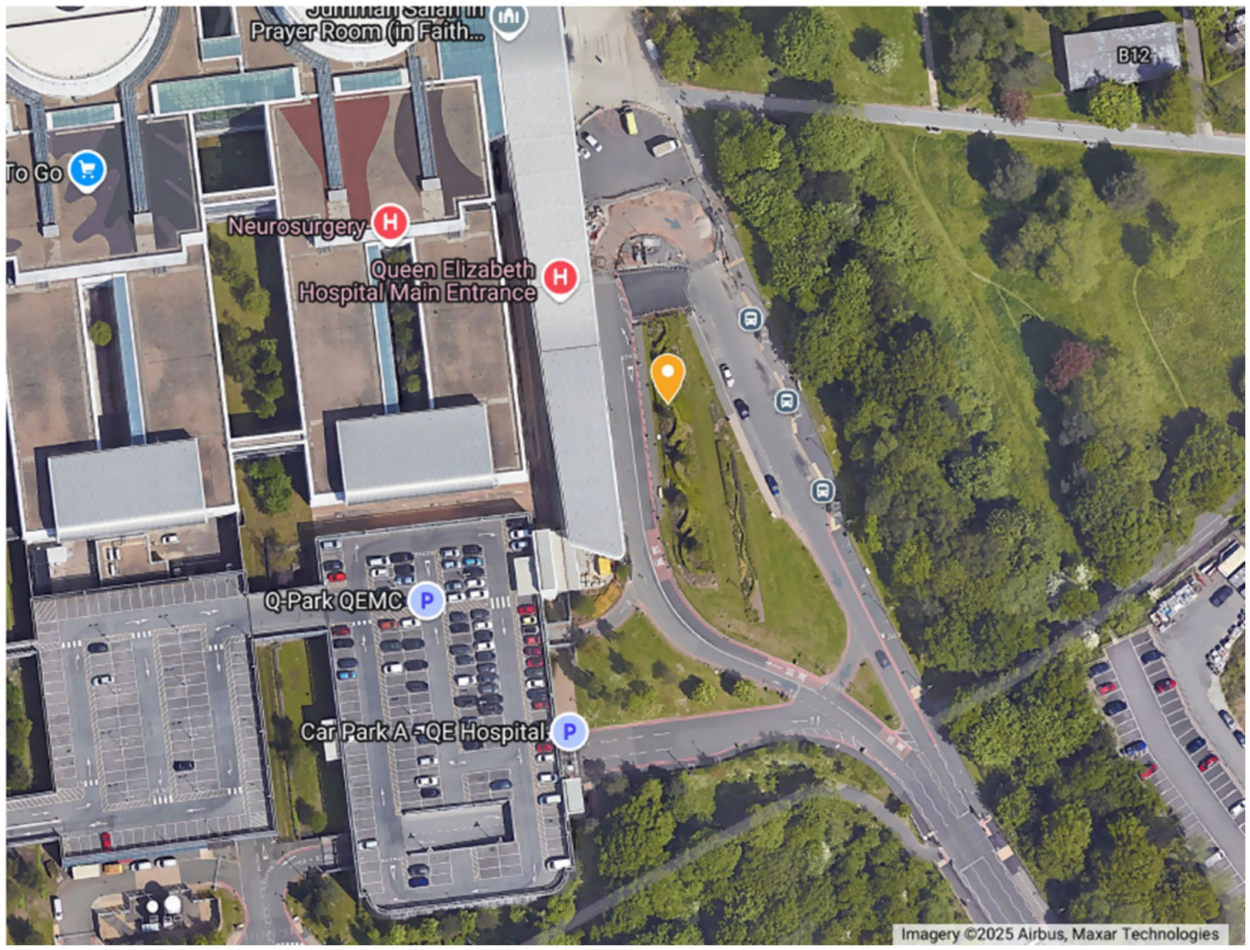
Satellite image of the QE Main Entrance loop with a yellow marker demonstrating the sampling location. Satellite map sourced from Google Maps (Imagery ©2025 Airbus, Maxar Technologies).

The location with a clearly distinguished difference between years for NO_2_ concentrations was QE Main Entrance. With the caveat that diffusion tubes offer indicative insight into concentrations at best, here data demonstrated a reduction between the concentrations in (7) and 2023 across all months. This is particularly encouraging for UHB as QE Main Entrance was highlighted as a hotspot in the (7) sampling campaign, consistently experiencing some of the highest concentrations for NO_2._ This location was chosen for sampling due the potential risk for personal exposures to and the tendency for idling vehicles and congestion. As shown in Figure 6, the area of sampling at QE Main Entrance is characterised by a short road loop, designed a drop off area for personal vehicles, taxis, some buses and non-emergency ambulance services. There is also an outdoor retail area, as well as seating, smoking and rest areas for staff, patients and visitors around the edge of the road loop and the entrance to one of the 3 multistorey carparks within the hospital campus is also located at one end of the loop.

The aforementioned traffic interventions (which reduced parking and introduced a bus lane to reduce congestion) may be a cause of the location-specific reduction in NO_2_ experienced here. Yet, this also provides insight into the further data required for intervention analysis and the feasibility of doing so at a hospital campus. To fully attribute changes to a specific intervention, data on traffic, meteorology, intervention dates and details would be useful, typically gathered by other authorities, highlighting the need for partnership working to enable robust evaluation. For example, Quintanilha et al. (63) were able to use passive sampling to detect reduction of NO_2_ along routes with bus lanes however this analysis was combined with traffic and meteorology data to support findings.

### 4.4 Limitations and transferability

This case study demonstrates the use of small form air quality monitoring within an acute tertiary hospital environment to support and evaluate actions intended to improve local air quality. Whilst this case study demonstrates successful operational deployment of sensor and diffusion tube technology for the purpose of in monitoring and detecting temporal changes in pollutant concentrations, it is important to recognise that the different meteorology, regional pollutant concentrations, location and design of healthcare settings require consideration in the design of future studies to evaluate air quality actions in healthcare settings.

This research was somewhat limited by the reduced period of follow up sampling in 2023 (4 months) compared to the initial year of sampling in 2020–2021. Above we disclosed the limitation of the follow up sampling period being only 4 months rather than a full year. Whilst there was interest from the healthcare professionals to conduct follow up sampling in 2023, there was reduced capacity and resources. Therefore we can only compare the impact of interventions across the April–July period, and cannot comment on changes in annual concentrations between 2020–2021 and 2023. Ideally, a follow up period would conduct with matched annual data, however this study shows that even with reduced capacity, healthcare settings can generate useful air quality insights.

The collaborative relationship between researchers at the University of Birmingham and the UHB NHS Foundation Trust was instrumental to the success of this project- enabling bilateral knowledge sharing, administrative and technical support and access to monitoring equipment and healthcare environments to sample in between the teams. With healthcare services experiencing high demand and competing priorities, the internal organisational human resource and capacity may be limited to support long-term monitoring activities. Moreover, further research is required to understand if similar successful partnerships can be achieved at locations which are not immediately located in close proximity to Higher Education or research institutions. Whilst this limits the generalisability of, it does show the importance and potential of collaboration between academia and healthcare delivery providers for creating impactful change for healthier environments.

## 5 Conclusion

With caveats for data quality (namely calibration methods, impact of meteorology, bias and error), these relatively low-cost methods for sampling in a healthcare setting were able to give insight into indicative pollutant concentrations. From diffusion tube data, a hotspot was detected as well as a statistically significant reduction in concentrations between the two sampling periods. This insight could be improved with extended diffusion tube sampling- this study only compares a few months of data in each year, but a full year’s data set for each period could also potentially allow for the insight into any seasonality in concentrations. Even without digital skills uptake or collaboration with researchers, diffusion tubes can provide insight that could support air quality awareness around healthcare settings. From this study, diffusion tubes are also currently more suited than small form sensors to supporting air quality monitoring at healthcare settings because of their smaller associated workloads in data management and the lack of need for specialist understanding, or continued funding for maintenance. There is a wealth of existing datasets that could enhance air quality understanding and support interventions within healthcare environments, but access to these is not always immediately available due to the varying stakeholders who own, manage and maintain healthcare settings and the surrounding area and operational data related to it. It is important to remember that collaborative relationships can take time to develop, and that for the best understanding of air quality on a hospital campus, these relationships need to establish beyond the initial contacts of the sustainability team to integrate all players who’s work impacts air quality (such as transport teams and estates management).

## Data Availability

The datasets presented in this article are not readily available because please contact authors regarding access to data. Requests to access the datasets should be directed to s.bartington@bham.ac.uk.
